# Mechanisms of weight gain associated with valproic acid: a scoping review

**DOI:** 10.1007/s00406-026-02295-x

**Published:** 2026-06-23

**Authors:** Darja Popkova, Frederike Fellendorf, Maj Vinberg, Deniz Ceylan, Aija Bukova-Zideluna, Eva Fleischmann, Nina Dalkner, Adelina Tmava-Berisha, Melanie Lenger, Mirko Manchia, Eva Reininghaus

**Affiliations:** 1 Department of Psychiatry and Psychotherapeutic Medicine, Medical University of Graz, Auenbruggerplatz 31, Graz, 8036 Austria; 2 The Early Multimodular Prevention and Intervention Research Institution (EMPIRI). Psychiatric Center, Copenhagen University Hospital, North Zealand, Denmark; 3 Department of Clinical Medicine, Faculty of Health and Medical Sciences, University of Copenhagen, Copenhagen, Denmark; 4 Department of Psychiatry, Koç University School of Medicine, Istanbul, Turkey; 5 Department of Psychiatry & Psychology, Mayo Clinic, Rochester, MN USA; 6 Institute of Public Health, Department of Public Health and Epidemiology, Riga Stradins University, Riga, Latvia; 7 Section of Psychiatry, Department of Medical Sciences and Public Health, University of Cagliari, Cagliari, Italy; 8 Unit of Clinical Psychiatry, University Hospital Agency of Cagliari, Cagliari, Italy; 9 Department of Pharmacology, Dalhousie University, Halifax, NS Canada

**Keywords:** Valproic acid, Weight gain, Lipid metabolism, Insulin resistance, Pharmacogenetics, Scoping review

## Abstract

**Introduction:**

Valproic acid (VPA), a widely prescribed mood stabilizer and antiepileptic drug, is frequently associated with metabolic side effects, particularly weight gain. Despite its clinical relevance, the range and diversity of mechanisms underlying VPA-associated weight gain remain incompletely mapped.

**Objectives:**

To map the breadth of evidence on mechanisms of VPA-associated weight gain, characterize the types of studies and populations investigated, and identify knowledge gaps to inform future research.

**Methods:**

This PRISMA-ScR-guided scoping review searched PubMed, Cochrane Library, Scopus, and Google Scholar for studies on VPA-weight gain mechanisms in adults. One reviewer charted data using a piloted form; two independently assessed quality (Newcastle-Ottawa Scale). Sources were grouped by mechanistic pathway with descriptive summaries and vote-counting.

**Results:**

Fifteen studies (*n* = 1,339 participants) were included, representing diverse designs: cross-sectional (*n* = 4), prospective observational (*n* = 3), genetic association (*n* = 2), and others. Evidence mapped to three primary mechanistic domains: hormonal dysregulation (like elevated insulin, leptin, insulin resistance, and other findings); genetic predispositions, including *CYP2C19* polymorphisms, *CRTC1* methylation, and other genetic variants; and lipid metabolism alterations (increased TC, LDL-C, TG, FFAs; decreased Apo A1; mixed HDL-C findings). Hormonal and lipid pathways are well-documented, while genetic mechanisms require validation in larger, standardized cohorts.

**Conclusions:**

This scoping review identifies a heterogeneous evidence base with well-established hormonal and lipid mechanisms and emerging genetic pathways. Key gaps include underrepresentation of bipolar disorder populations, limited longitudinal genetic studies, and lack of integrated frameworks combining hormonal, genetic, and metabolic data. Findings inform future research priorities and highlight the need for personalized monitoring strategies.

## Introduction

Valproic acid (VPA; glutamate transmission modulator, mechanism yet to be fully determined) is a widely used antiepileptic medication with a broad therapeutic profile, effectively managing partial and generalized seizures, treating acute mania, stabilizing mood in bipolar disorder, and reducing migraine frequency [1–3]. Despite its therapeutic efficacy, VPA use is often accompanied by metabolic side effects, with weight gain being one of the most prevalent and concerning [4]. Studies report that approximately 15–20% of patients on VPA experience weight gain, particularly during long-term use, raising significant health concerns [5].

Several risk factors have been identified as contributors to VPA-related weight gain, including serum levels exceeding 125 µg/mL, extended treatment duration beyond two to three months, and female sex [6–7]. In fact, some research suggests that even a modest increase in body weight of approximately two kg within the first month of VPA treatment may necessitate a reconsideration of the prescribed antiepileptic drug due to potential metabolic dysregulations [8]. However, despite this emerging evidence, a unified understanding of the mechanisms driving weight gain with VPA remains elusive. Therefore, the research question of this scoping review is: What mechanisms underlying weight gain are associated with the use of valproic acid?

Previous studies have shown that VPA-induced weight gain is multifactorial, reflecting the complex regulation of appetite at peripheral and central levels by various neuropeptides and cytokines, including adipose derived cytokines. Studies have proposed mechanisms such as hyperinsulinemia, insulin resistance, hyperleptinemia, and reduction of adiponectin as contributors to VPA-induced weight gain [8–13]. In addition, VPA has been suggested to promote weight gain by altering the expression of adipokine genes in the hypothalamus, affecting neuropeptides involved in central energy metabolism and leading to leptin and insulin resistance [8]. Another study reported elevated TG, malondialdehyde, and adiponectin—along with higher BMI—in bipolar disorder patients treated with VPA and lithium compared to healthy controls, indicating potential alteration in lipid peroxidation [11].

Mentioned mechanisms of metabolic alterations are especially crucial given the significant physical and mental health burden that weight gain can place on overweight and obese patients taking VPA. Alteration of somatic health under VPA-induced weight gain includes increased risks of dyslipidaemia, atherosclerosis, coronary syndrome, and diabetes mellitus [14–15]. Furthermore, weight gain adversely affects mental health, with numerous studies highlighting a bidirectional relationship between weight gain and mental health deterioration [16–17]. This relationship is especially concerning in adulthood, as weight gain has been associated with an increased risk of depression, along with heightened suicidality, hospitalization, and more severe symptoms like psychomotor agitation or retardation. This highlights the critical importance of weight management in supporting mental health [18].

While VPA-related obesity affects patients across multiple conditions like focal and generalized epilepsies, migraine, bipolar disorder provides a salient example where it exacerbates core psychiatric symptoms. Bipolar I disorder individuals with obesity experience a greater number of lifetime depressive and manic episodes, present with more severe and difficult-to-treat index affective episodes, and are more likely to develop affective recurrences, particularly depressive recurrences [19]. Addressing this adverse effect of VPA is of critical clinical importance, as it exacerbates long-term health risks associated with metabolic syndromes and negatively impacts patients’ quality of life.

Developing a deeper understanding of the mechanisms underlying VPA-induced weight gain is therefore essential, as it may improve medication management and guide future research on targeted prevention and treatment strategies.

Given this complex evidence landscape, a scoping review methodology appropriate for this research question because the evidence on VPA-associated weight gain mechanisms is diverse in study design (observational studies, genetic association studies, reviews), population (epilepsy, bipolar disorder, mixed psychiatric conditions), and outcome measurement (hormonal markers, genetic polymorphisms, lipid profiles). Rather than synthesizing effect sizes or comparing intervention effectiveness, this review aims to map the breadth of proposed mechanisms, characterize the nature of existing evidence, and identify knowledge gaps to inform future hypothesis-driven systematic reviews and primary research. This exploratory approach aligns with established scoping review frameworks [20–21].

##  Methods

### Literature search strategy

**Fig. 1:**
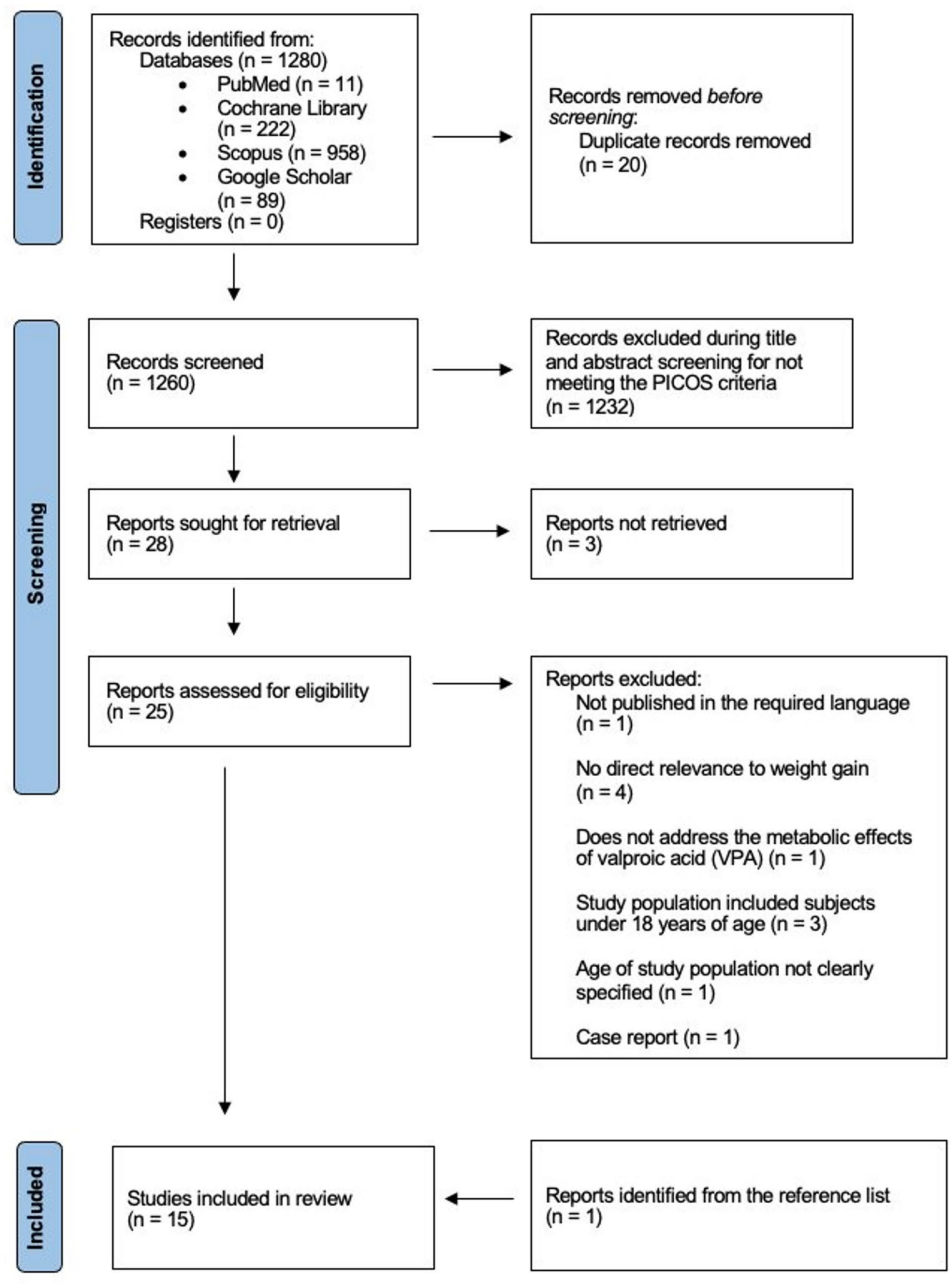
Study selection process following the PRISMA guidelines

This scoping review was conducted in accordance with PRISMA Extension for Scoping Reviews (PRISMA-ScR) guidelines [22]. The PRISMA checklist was used to ensure completeness and transparency of reporting. A comprehensive literature search was performed across multiple electronic databases, including PubMed, the Cochrane Library, Scopus, and Google Scholar (Fig. 1). The search strategy utilised a range of keywords, including “valproic acid” and synonyms, “weight gain” or “obesity”, “mechanism” or “metabolic effects”. Database-level filters were applied during the search to limit results to English-language publications, publications from 2014 to 2024, adult human populations, and eligible publication types. To identify additional relevant sources of evidence, the reference lists of included and cited articles were manually screened. Based on the PICO (Population, Intervention, Comparison, Outcome) model, the following descriptors were predefined (see Table 1), guiding the inclusion criteria and evidence synthesis for this scoping review.

**Table 1:** Research question according to the PICO model

First letter of component	Component	Descriptor
P	Patient/Problem of interest	Individuals receiving valproic acid treatment (aged > 18 years)
I	Intervention	Valproic acid
C	Comparison	Other treatments/no treatment
O	Outcome	Mechanism of weight gain

### Inclusion and exclusion criteria

Sources of evidence were included if they specifically addressed mechanisms of weight gain associated with VPA treatment and met the following criteria: publication in English, publication between 2014 and 2024, and inclusion of human participants aged 18 years and older. To capture the breadth of evidence, we included diverse study designs: observational studies (cross-sectional, prospective, retrospective cohort), genetic association studies, and review articles. Sources of evidence were excluded if they were case reports, case series, conference proceedings, animal or in vitro studies, or if they did not provide relevant information on mechanisms of weight gain or associated metabolic effects.

### Screening and selection process

The study screening and selection process is summarised in the PRISMA flow diagram (Fig. 1). Initially, records were identified through database searching. After removal of duplicate records, the remaining records were screened at the title and abstract level in accordance with the PICOS (Population, Intervention, Comparison, Outcomes, Study design) framework. Records not meeting the PICOS criteria were excluded. Study eligibility was determined through the systematic application of predefined inclusion and exclusion criteria, guided by the research question. Reasons for exclusion at the full-text stage were documented and are presented in the PRISMA flow diagram (Fig. 1).

A standardized data charting form was developed and piloted on three sources of evidence to ensure consistency and comprehensiveness. One reviewer charted data from all eligible sources, extracting: study design, sample size, population characteristics (diagnosis, sex distribution), VPA treatment duration, concomitant medications, proposed mechanisms, and key findings (hormonal markers, genetic polymorphisms, lipid alterations). Data items were selected to characterize the nature and extent of the evidence base rather than to extract effect sizes for meta-analysis. Variables included study design, population type (epilepsy, bipolar disorder, mixed psychiatric), treatment context (monotherapy vs. polytherapy), concomitant medications, and mechanistic pathways investigated (hormonal, genetic, metabolic). This approach enabled mapping of research trends, methodological diversity, and gaps in the literature. When information was unclear or missing, data were recorded exactly as reported in the original source, with no assumptions made.

### Data synthesis

Due to heterogeneity in study design, populations, treatment duration, and outcome measures, meta-analysis was not conducted. Included sources were grouped by proposed mechanistic pathway (hormonal dysregulation, genetic predisposition, lipid metabolism alterations) based on data charted from each study. Descriptive summaries mapped patterns, consistencies, and divergences across studies. Vote-counting based on statistical significance (Table 4) summarized evidence consistency.

### Critical appraisal

Although quality appraisal is optional in scoping reviews, we assessed methodological quality using the Newcastle–Ottawa Scale (NOS) for 13 primary observational studies [23]. Two reviewers independently evaluated these studies, resolving discrepancies through discussion. Review articles were excluded from this assessment. The quality ratings helped interpret our findings but did not affect study inclusion, which follows standard scoping review practice.

## Results

A comprehensive search identified fifteen sources of evidence investigating mechanisms underlying weight gain associated with VPA treatment (Table 2). Based on study design, this review included fourteen sources of evidence [24–37] and one cross-referenced article [38], representing a range of methodological approaches: four cross-sectional studies [27–30, 37], three prospective observational studies [25–27, 33], two genetic association studies [24, 32], one comparative prospective study [35], one longitudinal observational study [29], one comparative observational study [36], two review articles [31, 34], and one retrospective longitudinal study [38]. The populations examined consisted of human patients, predominantly individuals with epilepsy, alongside patients with bipolar disorder, schizoaffective disorder, and other conditions, receiving either VPA monotherapy or polytherapy. In total, 12 sources of evidence described VPA therapy in patients with an epilepsy diagnosis, and only three of them presented patients with psychiatric diagnoses such as bipolar disorder or schizoaffective disorder, represented as a group of conditions (Table 2).

In total, the sources of evidence included 1,339 participants, with at least 906 receiving VPA monotherapy and the remainder receiving VPA in combination with other antiepileptic drugs, antipsychotics, mood stabilizers, or antidepressants (Table 3). Some sources of evidence provided additional demographic information: for instance, Islamiyah et al. (2022) [28] included 40 patients (17 males, 23 females), and Hamed et al. (2016) [27] exclusively enrolled 66 female patients. However, sex distribution was not consistently reported across all sources of evidence. Two studies—Shnayder et al. (2023) [34] and Mangge et al. (2019) [31]—did not report exact sample sizes and were excluded from the participant count.

**Table 2:** Studies investigating mechanisms of weight gain under valproic acid treatment.

Study	Study Design	Sample Size	Population	Treat-ment duration	Con-comitant medication	Mechanism of weight gain
Li et al., 2015	Prospective observational cohort study	212	PWE	6 months	no	*LEPR*, *ANKK1*, *AMPK SNPs* linked to VPA-induced WG
Hamed et al., 2016	Cross-sectional	190	PWE + matched controls	6 to 20 years	no	Insulin, leptin and lipid metabolism alterations
Noai et al., 2016	retrospective longitudinal	178	PWE	22 years	yes	*CYP2C19* polymorphisms
Sidhu et al., 2017	comparative prospective study	66	newly diagnosed or untreated F PWE	1 year	no	Insulin, adiponectin and HOMA-IR alterations
Bai et al., 2018	genetic association investigation	225	Chinese Han PWE	1 year	yes	*CD36* and *PPARγ* polymorphisms
Mangge et al., 2019	review article	not specified	BD	not specified	no	Inhibition of Histone Deacetylase
Delacrétaz et al., 2019	prospective observational study	78	PD, SD, BD, DD, OD, a.o.	1 month	yes	methylation changes in the *CRTC1* gene
Li et al., 2019	Cross-sectional observational study	6	PWE on VPA + HI	6 months	not stated	lipid metabolism alterations (TG)
Delacrétaz et al., 2021	prospective observational study	78	PD, BD, DD, OD and a.o.	1 year	yes	altered lipid metabolism (HDL-C levels)
Balachandran et al., 2021	prospective genetic association	108	PWE	6 months	no	*CYP2C19* polymorphisms
Şimşek et al., 2021	comparative observational study	60	PWE	*21 months	no	Insulin resistance, carnitine and adiponectin
Islamiyah et al., 2022	cross-sectional study	40	PWE	6 months	no	*CYP2C19* polymorphisms
Yaryari et al., 2022	cross-sectional longitudinal	66	PWE (TC) + HI	*5.64 years	no	Increased asprosin level
Shnayder et al., 2023	narrative review	not specified	PWE, BD, S, SD, OPD	not specified	not mentioned	insulin, leptin and lipid metabolism alterations
Sarangi et al., 2024	prospective comparative study	92	PWE	6 months	no	insulin, leptin, and adiponectin alterations

*AMPK* Adenosine Monophosphate-Activated Protein Kinase, *ANKK1* gene Ankyrin Repeat and Kinase Domain Containing 1, a.o. and others, BD bipolar disorder, *CD36* Cluster of Differentiation 36, *CRTC1* gene CREB Regulated Transcription Coactivator 1, *CYP2C19* = Cytochrome P450 2C19 enzyme, *DD* Depressive Disorder, *F* female, *HDL-C* High-Density Lipoprotein Cholesterol, *HI* Healthy Individuals, *HOMA-IR* Homeostatic Model Assessment of Insulin Resistance, *LEPR* Leptin Receptor, *OD* Organic Disorders, *OPD* Other Psychotic Disorders, *PD* Psychotic Disorders, *PPARγ* – Peroxisome Proliferator-Activated Receptor Gamma, *PWE* Patient with Epilepsy, *S* Schizophrenia, *SD* Schizoaffective Disorders, *SNPs* Single Nucleotide Polymorphisms, *TG* Triglyceride, *VPA* Valproic Acid, *WG* Weight Gain; *mean

**Table 3:** Concomitant medication use with valproic acid

Medication context	Number of studies		
VPA as monotherapy	11		
VPA and co-medications	4		
**Medication group used with VPA**		Co-medication	Number of studies reported
**Antiepileptics**			
		Carbamazepine	1
		Clonazepam	1
		Ethosuximide	1
		Lamotrigine	1
		Levetiracetam	2
		Oxcarbazepine	1
		Topiramate	1
		Zonisamide	1
**Antipsychotics**			
		Amisulpride	2
		Asenapine	2
		Clozapine	1
		Haloperidol	1
		Olanzapine	1
		Quetiapine	2
		Risperidone	2
		Zuclopenthixol	2
**Mood Stabilizers**			
		Lithium	2
**Antidepressant**			
		Mirtazapine	2

This scoping review mapped several mechanisms potentially contributing to weight gain in patients receiving VPA-therapy (Table 4). These mechanisms were categorized into three main pathways: hormonal dysregulation, genetic predispositions, and alterations in lipid metabolism.

### Hormonal dysregulation

The first pathway, hormonal dysregulation, emerged as the most frequently described mechanism of VPA-associated weight gain, reported in six sources of evidence [27, 33–37]. Four sources of evidence specifically described insulin resistance [33–36], three reported elevated leptin levels [33–35], three noted reduced adiponectin [33, 35–36] and one study identified increased asprosin levels [37].

Hormonal alterations, including elevated insulin, increased leptin, and decreased adiponectin, were consistently reported in sources of evidence assessing VPA-associated weight gain. Sarangi et al. (2024), Sidhu et al. (2017), Hamed et al. (2016), and Shnayder et al. (2023) all demonstrated significantly increased insulin levels or higher Homeostasis Model Assessment of Insulin Resistance (HOMA-IR) scores, with no conflicting findings [29, 35–37]. Leptin elevations were similarly reported by Sarangi et al. (2024) [33] and Hamed et al. (2016) [27], with Shnayder et al. (2023) [34] supporting its role in weight gain; however, Sidhu et al. (2017) [35] noted leptin activation was comparable to obese controls, suggesting levels may reflect body fat mass rather than a direct drug effect. Findings on adiponectin were mixed: Sidhu et al. (2017) [35] reported significant decreases in adiponectin levels associated with VPA treatment observed in comparison to the lamotrigine (glutamate voltage-gated sodium channel blocker) group (*p* < .001, *p* < .05), whereas Şimşek et al. (2021) [36] observed no differences between VPA-, carbamazepine (glutamate voltage-gated sodium and calcium channel blocker)-treated, and healthy controls.

(*p* = .780). Yaryari et al. (2022) [37] further identified significantly elevated fasting serum asprosin in VPA-treated patients versus controls (*p* < .001) and lamotrigine-treated or untreated groups (*p* < .01), suggesting a novel hormonal pathway contributing to weight gain. However, no covariate adjustment (e.g., for BMI) was performed to determine causality independent of baseline differences across the four groups.

### Genetic predisposition

The second pathway, genetic polymorphisms and gene regulation, was examined in seven sources of evidence addressing genetic predispositions as contributors to weight gain in VPA-treated patients [24–25, 28–29, 31–32, 38]. Three sources of evidence investigated *CYP2C19* polymorphisms, focusing on intermediate (*1/*2: one functional *1 and one defective *2 allele) and poor (*2/*2: two defective *2 alleles*)* metabolizers: Balachandran et al. (2021) and Noai et al. (2016) [32, 38] reported significant associations, while Islamiyah et al. (2022) [28] found no significant relationship. Since valproic acid undergoes complex metabolism via UGT1A/UGT2-mediated glucuronidation, mitochondrial β-oxidation, and CYP-mediated ω-oxidation Ito et al. (1990) [39], variation in enzymes such as CYP2C19 may partly account for interindividual differences in VPA-induced weight gain. Additional mechanisms included epigenetic changes, with Mangge et al. (2019) proposing histone deacetylase (*HDAC*) inhibition as a factor influencing metabolic gene expression [31], and Delacrétaz et al. (2019) reporting significant methylation alterations at three CpG sites in the CRTC1 gene associated with ≥ 5% weight gain (*p* = .02) [25]. Bai et al. (2018) identified *CD36 rs1194197 C*, *CD36 rs7807607 T*, and *PPARγ rs10865710* C alleles as protective against VPA-induced obesity (all *p* < .05, Bonferroni corrected) [24].

**Table 4:** Summary of mechanisms of weight gain under treatment with valproic acid

Mechanisms	Number of studies	Mechanisms found significant (*)	Mechanism found not significant (-)
Insulin resistance	4	4	0
Increased leptin	3	3	0
Decreased adiponectin	3	2	1
increased total and LDL cholesterol	2	2	0
Increased HDL-C	2	2	0
Increased TG	2	2	0
Decreased Apo A1	1	1	0
Increased FFAs	1	1	0
Decreased carnitine	1	0	1
*CYP2C19* polymorphisms *1/2 (IM)* and *2/2 (PM)*	3	2	1
Increased asprosin	1	1	0
Inhibition of Histone Deacetylase	1	1	0
Methylation changes in the *CRTC1* gene	1	1	0
*CD36* and *PPARγ* polymorphisms	1	1	0
*LEPR*, *ANKK1*, *AMPK SNPs* polymorphism	1	1	0

*AMPK* AMP-Activated Protein Kinase, *ANKK1* Ankyrin Repeat and Kinase Domain Containing 1, Apo A1 Apolipoprotein A1, *CD36* Cluster of Differentiation 36, *CRTC1* gene CREB Regulated Transcription Coactivator 1, *CYP2C19* Cytochrome P450 Family 2 Subfamily C Member 19, FFAs Free Fatty Acids, HDL-C High-Density Lipoprotein Cholesterol, *IM* Intermediate Metabolizer, *LDL* Low-Density Lipoprotein, *LEPR* Leptin Receptor, PM = Poor Metabolizer, *PPARγ* = Peroxisome Proliferator-Activated Receptor Gamma, SNPs = Single Nucleotide Polymorphisms, TG = Triglycerides.

### Lipid metabolism

The third pathway involves alterations in lipid metabolism, investigated in four sources of evidence examining VPA-induced lipid changes and mechanisms contributing to fat accumulation [26–27, 30, 36]. Reported alterations included elevated total cholesterol (TC), low-density lipoprotein cholesterol (LDL-C), triglycerides (TG), and free fatty acids (FFAs), alongside decreased high-density lipoprotein cholesterol (HDL-C) and apolipoprotein A1 (Apo A1) levels. While most findings were consistent, results for HDL-C were mixed: Delacrétaz et al. (2021) reported a significant decrease in HDL-C after one month of VPA therapy (*p* = .02), with 54% of patients experiencing ≥ 5% reductions [26], whereas Hamed et al. (2016) found no significant difference compared to controls (*p* > .05) [27]. Li et al. (2019) additionally demonstrated reduced Apo A1 levels in VPA-treated patients (*p* = .033) [30]. Şimşek et al. (2021) also observed lower carnitine levels in VPA-treated patients compared to carbamazepine (CBZ)-treated patients and healthy controls, but this difference was not statistically significant (*p* = .163) [36].

## Discussion

### Summary of evidence

This scoping review mapped the breadth and nature of evidence on mechanisms underlying VPA-associated weight gain, identifying three primary domains: hormonal dysregulation, genetic predispositions, and lipid metabolism alterations. The evidence base is characterized by methodological diversity (cross-sectional, prospective, genetic association studies) and population heterogeneity (epilepsy, bipolar disorder, mixed psychiatric conditions). Our findings reveal well-established hormonal and lipid pathways alongside emerging but underexplored genetic mechanisms, highlighting key knowledge gaps that warrant further investigation.

Hormonal dysregulation emerged as the most consistently reported mechanism of VPA-associated weight gain. Multiple sources of evidence demonstrated significant increases in insulin levels and HOMA-IR scores, indicating insulin resistance even in patients without overt obesity. One plausible mechanism proposed in previous studies contributing to hyperinsulinemia and insulin resistance is oxidative stress in pancreatic beta-cells Belcastro et al. (2013) [40]. VPA induced oxidative stress results from imbalance between reactive oxygen species production and antioxidant defenses Tong et al. (2005) [41], leading to lipid peroxidation and cellular dysfunction, which contributes to metabolic dysregulation and weight gain Di Domenico et al. (2019) [42]. Leptin levels were similarly elevated, suggesting impaired satiety signaling. Findings on adiponectin were inconsistent, with some studies reporting reduced levels under VPA therapy, warranting further investigation. Additionally, asprosin—a glucogenic adipokine secreted by white adipose tissue and first described by Romere et al. (2016) [43]—has been implicated in obesity-related metabolic disturbances and may represent a novel biomarker of VPA-induced metabolic risk. Future studies should investigate the role of asprosin, employing covariate analysis to determine the causal relationship between VPA treatment and weight gain, and evaluate its predictive potential for metabolic complications in VPA-treated patients alongside established hormonal markers.

Genetic predisposition is an emerging area of research in VPA-induced weight gain, with *CYP2C19* polymorphisms being the most frequently investigated. However, evidence remains inconclusive, and larger, standardized studies are needed to validate these associations, as current research often relies on small, heterogeneous cohorts (e.g., Islamiyah et al. (2022), *n* ≈ 40) [28]. Other genetic mechanisms, including *CRTC1* methylation changes and polymorphisms in *LEPR*, *ANKK1*, and *AMPK* genes, also show promise in elucidating VPA-related metabolic disturbances. Expanding research in these areas may establish genetic markers that could guide individualized VPA therapy based on patient-specific genetic profiles.

VPA therapy has been linked to adverse lipid changes, including increased TC, LDL-C, TG, and FFAs, along with reduced HDL-C and Apo A1 levels [26–27, 29, 33]. While most findings were consistent, HDL-C results were mixed: Delacrétaz et al. (2021) reported significant HDL-C reductions after one month of treatment [26], whereas Hamed et al. (2016) found no difference compared to controls [27]. Li et al. (2019) further reported a significant Apo A1 decrease, relevant given its central role in HDL particles [29]. Future studies should use standardized methodologies, clearly define treatment durations, control for baseline lipid status and co-medications, and apply longitudinal designs to better characterize VPA-induced lipid alterations.

### Limitations

As a scoping review, this study prioritized comprehensive mapping over detailed quality filtering or effect estimation. We did not exclude studies based on methodological quality, which may introduce variability in reported mechanisms. Scoping reviews do not synthesize effect sizes or grade evidence certainty (e.g., GRADE), limiting causal inferences. Our optional NOS assessments provided interpretive context without specific ratings reported.

Another limitation of this scoping review is the heterogeneity of study populations and co-medication use, introducing significant bias. Diverse patient groups, including those with bipolar disorder and epilepsy, likely reflect distinct metabolic profiles and varying susceptibility to weight gain. Studies have shown elevated leptin and insulin levels in euthymic bipolar disorder patients, potentially driven by both medication effects (VPA and co-administered drugs) and mood-related insulin dysregulation [3]. Co-medication variability further complicates interpretation, as antipsychotics such as olanzapine (dopamine, serotonin receptor antagonist (D2, 5-HT2)), asenapine (dopamine, serotonin, noradrenaline receptor antagonist (D2, 5-HT2, NE alpha-2)), risperidone (dopamine, serotonin, noradrenaline receptor antagonist (D2, 5-HT2, NE alpha-2)), aripiprazole (dopamine, serotonin receptor partial agonist (D2, 5-HT1A), and quetiapine (dopamine, serotonin, noradrenaline MM; receptor antagonist (D2, 5-HT2) and reuptake inhibitor (NET)(metabolite)) are independently associated with weight gain [44]. Treatment duration also varied widely (6 months to 22 years), with some studies reporting progressive visceral fat accumulation and weight gain of ~ 1 kg/year under long-term VPA therapy [45]. These factors may significantly influence observed metabolic effects and confound conclusions.

Screening and data charting were performed by a single reviewer, introducing potential selection bias. Additionally, restricting to English-language publications (2014–2024) may have missed relevant non-English or earlier mechanistic studies.

### Implications and future directions

Our findings support routine monitoring of hormonal markers (insulin, leptin) and lipid profiles (TC, LDL-C, TG, FFAs) in VPA therapy for early intervention, particularly in high-risk bipolar patients facing compounded metabolic and mental health risks [46]. Future research should validate genetic predictors like *CYP2C19* polymorphisms in larger cohorts, develop integrated hormonal-genetic-metabolic frameworks, and design personalized prevention strategies. Further it is essential investigation of biomarkers such as asprosin and adiponectin using covariate analysis (e.g., ANCOVA adjusting for BMI) to establish causality between VPA treatment and weight gain. These steps address key gaps in longitudinal data, underrepresented populations, and mechanistic synthesis.

## Conclusions

This scoping review maps evidence on VPA-associated weight gain mechanisms, identifying hormonal dysregulation, genetic predispositions, and lipid alterations as primary pathways. Hormonal (insulin, leptin) and lipid markers (TC, LDL-C, TG, FFAs) show consistent associations, while genetic findings—particularly *CYP2C19* polymorphisms—need validation in larger cohorts. Key gaps include bipolar patient underrepresentation, few longitudinal genetic studies, and absent integrated models. These findings guide key research priorities, including validating genetic predictors across diverse populations, testing emerging biomarkers like asprosin and adiponectin, developing multi-pathway frameworks, and targeting high-risk groups. Clinically, routine monitoring of hormones and lipids can enable early intervention and support personalized VPA management.

## Data Availability

No datasets were generated or analysed during the current study.
